# Simultaneous bilateral traumatic clavicle fractures: incidence, characteristics, and surgical outcomes

**DOI:** 10.1186/s12891-023-06228-w

**Published:** 2023-02-10

**Authors:** Dongxu Feng, Wuqiang Jiang, Xiaomin Kang, Yuxuan Jiang, Yangjun Zhu, Jun Zhang

**Affiliations:** 1 Department of Orthopaedic Trauma, Hong Hui Hospital, Xi’an Jiaotong University School of Medicine, Xi’an, Shaanxi China; 2 Center for Translational Medicine, The First Affiliated Hospital of Xi’an Jiaotong University, Xi’an, Shaanxi China

**Keywords:** Bilateral clavicle fractures, Plate, Surgery, Incidence, Characteristics, Surgical outcomes

## Abstract

**Background:**

Although clavicle fractures are common injuries in adults, simultaneous bilateral clavicle fractures are rarely reported. The present report describes 13 patients with simultaneous bilateral traumatic clavicle fractures who were treated with surgical management and followed for more than 12 months postoperatively.

**Methods:**

This retrospective chart review involved skeletally mature patients with traumatic clavicle injuries. Patients with bilateral clavicle fractures who were followed up for at least 12 months after surgery were included. Data regarding the patients’ demographics, injury characteristics, fracture classification, comorbidities, concomitant injuries, and treatment strategies were collected. Each displaced fracture was managed with open reduction and internal fixation. Postoperative follow-up included radiographs for assessment of bone union; calculation of the Constant–Murley score for shoulder function; administration of the Disability of the Arm, Shoulder, and Hand questionnaire for upper limb function; determination of the visual analogue scale score for pain; and assessment of complications.

**Results:**

From October 2013 to November 2021, 15 patients (10 men, 5 women) were diagnosed with bilateral clavicle fractures among 1542 patients with clavicle injuries (overall incidence of 1.0%). Of these 15 patients, this study included 13 patients (8 men, 5 women; mean age, 38.3 ± 15.3 years) who were followed up for more than 12 months postoperatively. Among the 13 patients, 10 (77.0%) had associated concomitant injuries, and 25 sides were fixed with internal plate fixation. After a follow-up period of 29.9 ± 28.5 months, all fractures achieved bone healing. Eleven patients attained excellent shoulder function on both sides and returned to their pre-injury daily activities, and the remaining two patients had unilateral shoulder dysfunction. No complications occurred.

**Conclusions:**

Bilateral clavicle fractures are extremely rare and associated with polytrauma. Open reduction and internal fixation is recommended for such patients, especially those with severe chest injuries, because osteosynthesis of the clavicle can improve respiratory function and reduce the duration of functional disability.

## Background

The clavicle a double-curved S-shaped bone, and it is the only long horizontal bone connecting the axial and upper girdle bones. Clavicle fractures are very common injuries with an incidence of 30 per 100,000 persons and represent 2.6% to 4.0% of all fractures [1]. Approximately 70% to 80% of clavicle fractures occur in the middle third of the clavicle [2]. By contrast, lateral and medial clavicle fractures account for 28% and 3%, respectively, of all clavicle fractures [3]. On the other hand, mid-shaft clavicle fractures most often occur by high-energy injuries in young patients, distal clavicle fractures most often occur by ground-level falls in advanced-age patients with osteoporosis, and medial clavicle fractures occur more often by high-energy trauma or trauma with multiple injuries [4].

The primary function of the clavicle is to act as a strut for the scapula to suspend the upper limb away from the thorax, thus allowing the extensive range of movement exhibited by the upper limb. Stable or non-displaced medial and distal clavicle fractures can be treated conservatively; the nonunion rate can reach 10% to 23% after conservative treatment for displaced midshaft clavicle fractures. Open reduction and internal fixation (ORIF) is mostly used for adults with displaced clavicle fractures and can achieve promising results [1]. Surgery is associated with a lower risk of non-union and provides a shorter time to return to work and better limb function. Operative treatment consists of intramedullary fixation and internal plate fixation, with the latter mostly used in the clinical setting.

Although fractures of the clavicle are among the most frequent fractures in adults, simultaneous traumatic bilateral clavicle fractures in adults are reported more rarely [5–8]. Based on the few published reports, bilateral clavicle fractures represent less than 0.5% of all clavicle fractures [6, 9]. In this study, we analyzed the data of 13 patients with simultaneous bilateral clavicle fractures who were followed up for more than 12 months after operative management.

## Materials and methods

### Study design and participants

The clinical data of all skeletally mature patients with traumatic clavicle injuries treated in our institute from October 2013 to November 2021 were retrospectively reviewed. The inclusion criterion was skeletally mature patients with bilateral clavicle fractures treated by ORIF with a follow-up of more than 12 months. The exclusion criteria were an age of less than 18 years, a unilateral clavicle injury, and bilateral clavicle injuries with dislocation of the sternoclavicular joint or acromioclavicular joint on one side. The patients’ charts and radiographs were collected from the institution’s electronic medical record system and reviewed for the patients’ demographic data, injury characteristics, fracture classification, comorbidities, concomitant injuries, and treatment. The fractures were divided into medial-third, middle-third (shaft), and distal-third fractures according to the Allman classification [10].

This study was conducted according to the Declaration of Helsinki and approved by the Ethics Committee of Hong Hui Hospital (NO.202208002). Written informed consent was obtained from individual or guardian participants.

### Surgical technique

The aim of operative management was to provide an optimal outcome for each individual patient. The surgical indications in this study were displaced clavicle fractures that could not be managed by closed reduction, re-displaced fractures after closed reduction, and fractures associated with concomitant injuries in the ipsilateral upper extremity that required early function exercises after surgery. Each operation was performed by well-trained orthopedic surgeons under general anesthesia. The patient was placed on a translucent orthopedic table in the supine position with a bump placed between the two scapulae, allowing the injured arm to be in a mobile position. One dose of a cephalosporin was given at induction and continued for 2 days postoperatively.

**Fig. 1:**
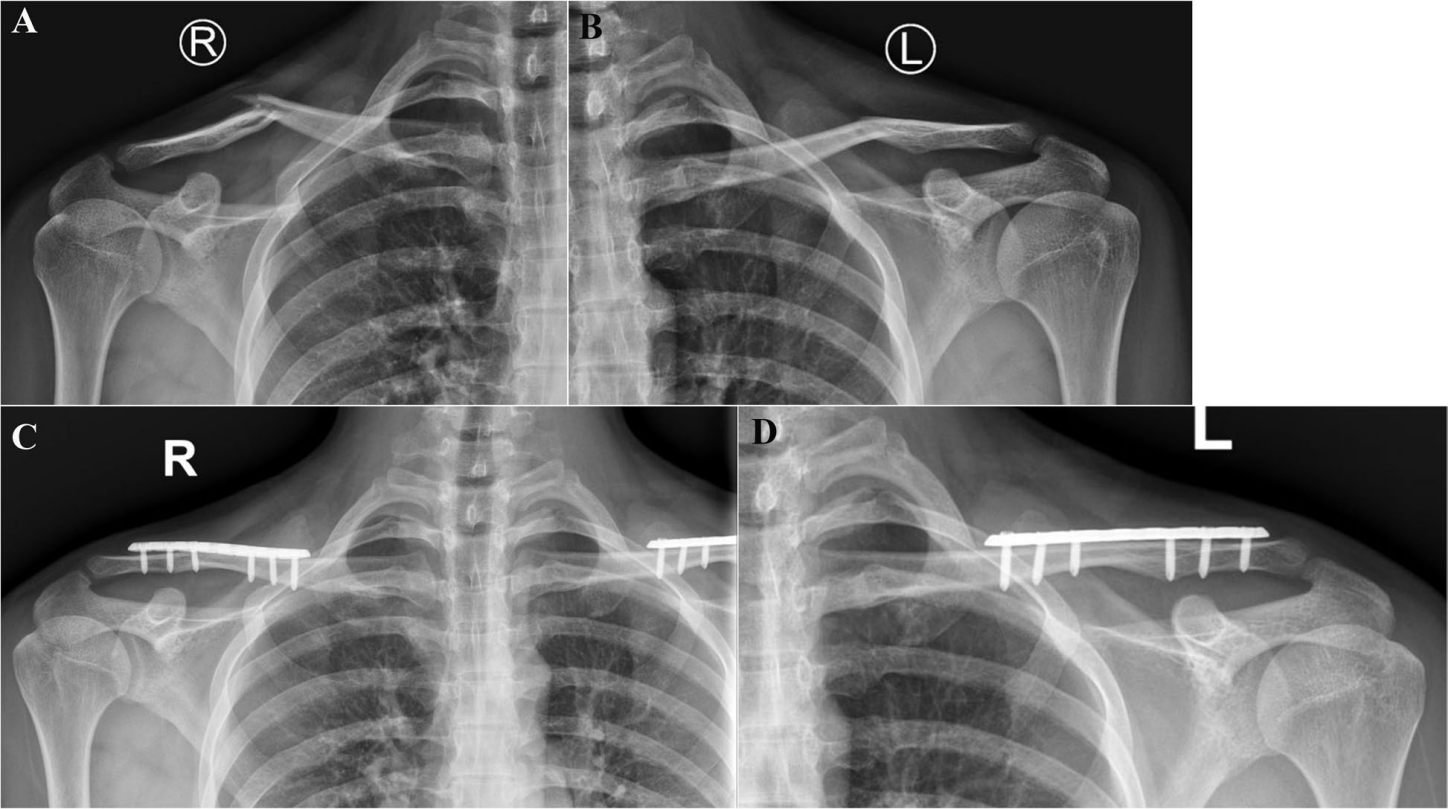
**A**, **B** Radiographs of an 18-year-old male patient showed bilateral displaced mid-clavicle fractures. **C**, **D** Follow-up radiographs at 6 months showed healing of the bilateral clavicle fractures after internal plate fixation on both sides

**Fig. 2:**
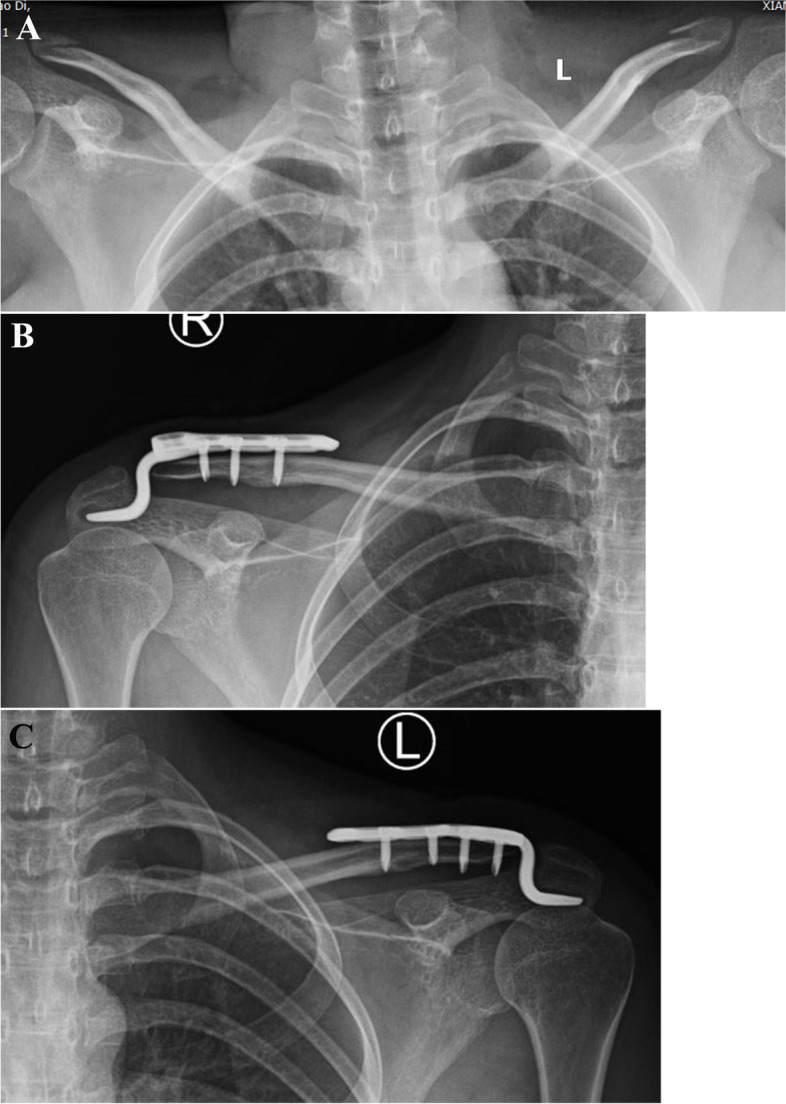
Radiographs of a 28-year-old woman who sustained bilateral clavicle fractures caused by a car accident; she also had a right radial shaft fracture, left ulnar olecranon fracture, and lumbar fracture. **A** Preoperative radiograph showed bilateral displaced distal clavicle fractures. The fractures on both sides were fixed with a hook plate. **B**, **C** Radiographs taken at the 1-year follow-up showed that the bilateral fractures had achieved bone union without implant migration

A longitudinal incision centered over the clavicle fracture was made. The platysma was released, and the supraclavicular nerve was identified and protected. After debridement, the fracture was directly examined and reduced with bone clamps and temporarily fixed by Kirschner wires. A lag screw was then placed across the fracture line before plating, if possible. For middle-shaft fractures of the clavicle, a 3.5-mm plate was contoured to fit along the superior edge of the clavicle; screws were then inserted from superior to inferior, ensuring placement of at least three screws with six cortical fixations at each fracture end (Fig. 1). When a superior plate was used for lateral or medial clavicle fractures, the technique was similar to superior plating for middle-shaft fractures. When a hook plate was used for lateral clavicle fractures, the hook was first inserted into the subacromial space, and the other end was then fixed on the superior part of the clavicle (Fig. 2). When a hook plate was used for a medial clavicle fracture, the fracture fragments were temporarily fixed and a gap was bluntly created between the medial head of the clavicle and the first rib at the posterior dorsal-osteal face of the sternal manubrium. The hook was then inserted into the retrosternal space, and the other end was fixed on the clavicle. Meticulous attention was mandatory to preserve the periosteum and avoid injury to the subclavian vessels and lungs during the whole operation. Fracture reduction, plate positioning, and screw length were verified by intraoperative X-ray examination. Finally, the surgical wound was closed in layers.

### Postoperative treatment and statistical analysis

The patients were immobilized in a sling for 3 to 4 weeks postoperatively. Codman’s pendulum exercises were then gently started, and the patients gradually began to perform passive shoulder functional exercises. The patients were also encouraged to use the arm but to avoid strengthening exercises until 3 months postoperatively, and they were permitted to return to their regular activity at 6 months postoperatively. Plate removal was not routinely performed unless hardware irritation occurred. Hook plate removal was encouraged 12 months after surgery.

The patients were encouraged to undergo follow-up at the authors’ institute at 1, 2, 3, and 6 months postoperatively and every 6 months thereafter or until full bone healing. Plain radiographs were taken to evaluate the bone healing status, joint congruency, and hardware failure or migration. A computed tomography scan was performed if there was not enough evidence of the bone healing status on X-rays. Clinical evaluation consisted of calculating the Constant–Murley score for shoulder function (higher scores indicated higher levels of shoulder function); administering the Disability of the Arm, Shoulder, and Hand questionnaire for upper limb function (lower scores indicated higher levels of limb function); and determination of the visual analogue scale score for pain (higher scores represented higher levels of pain) [11]. Fracture union was defined as evidence of at least three of four healed cortices across the fracture site. Any complications were also recorded. The bone healing status, complications, and functional outcome scoring at the end of follow-up were evaluated. Demographic characteristics are presented as mean and standard deviation.

## Results

**Fig. 3:**
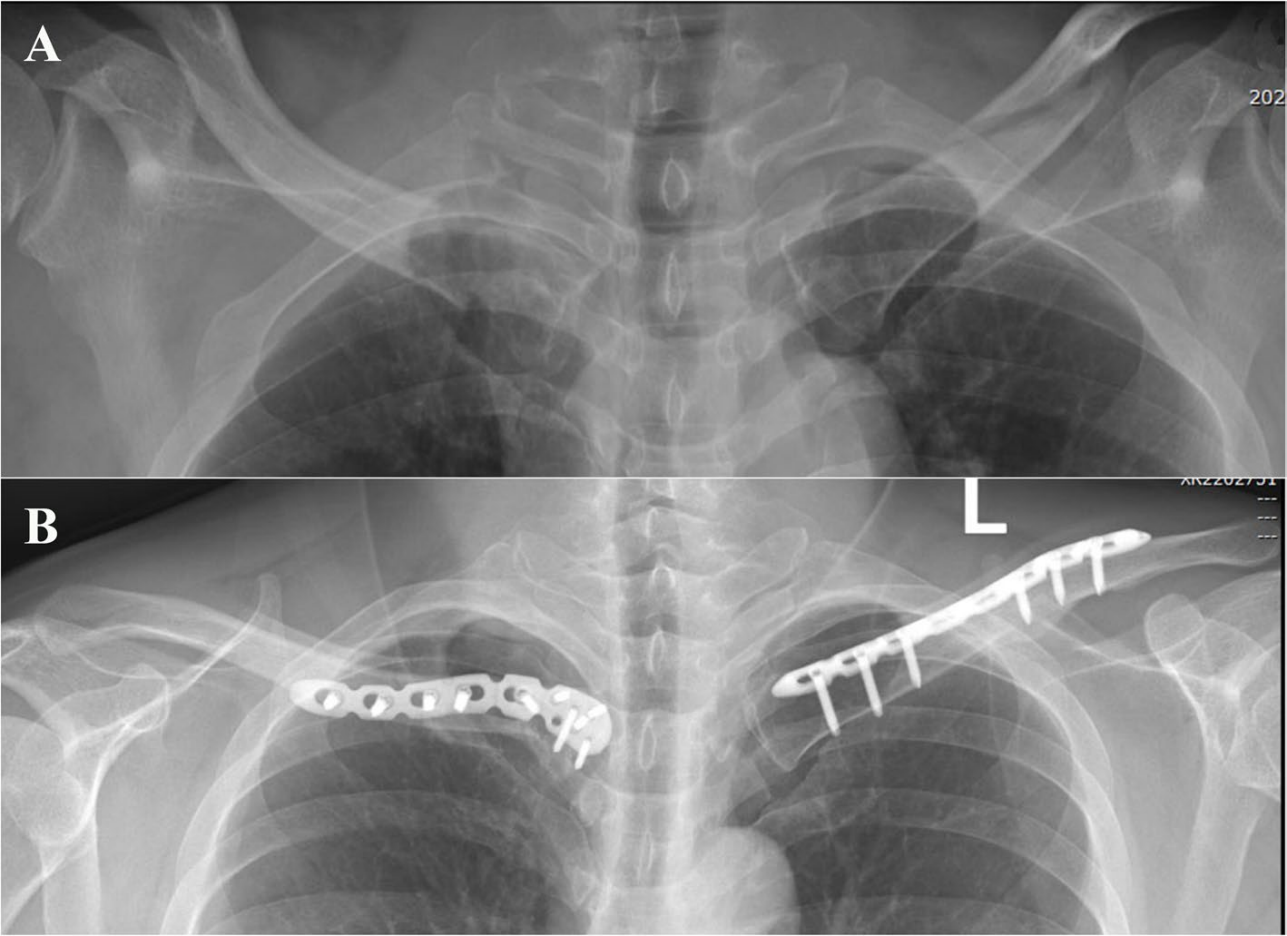
**A** Preoperative radiograph of a 53-year-old man showed a right intra-articular medial clavicle fracture and left mid-shaft clavicle fracture. **B** Radiograph 14 months postoperatively showed solid bone union on both sides after inverted distal clavicle plate fixation for the right clavicle fracture and reconstructive plate fixation for the left mid-clavicle fracture

**Fig. 4:**
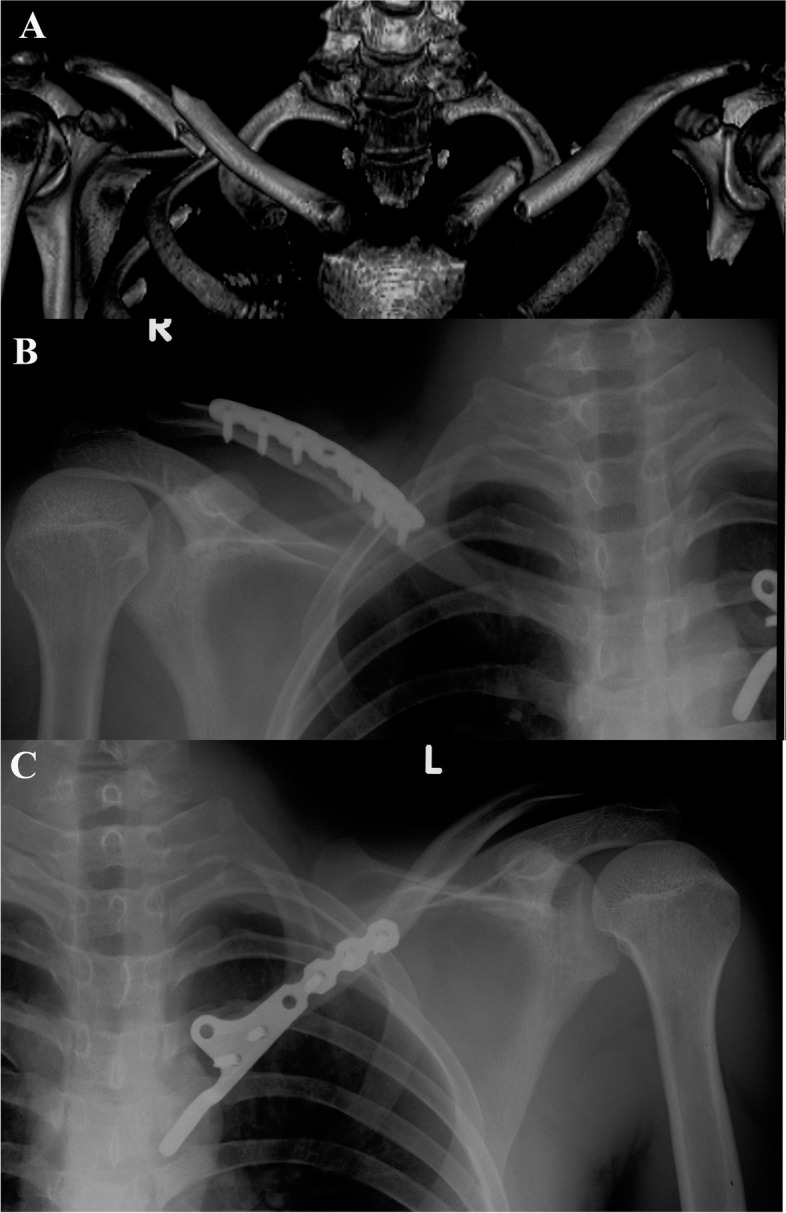
**A** Three-dimensional computed tomography reconstruction showed a displaced right middle-shaft clavicle fracture and a displaced left medial clavicle fracture. Both fractures were repaired by internal plate fixation. **B**, **C** An immediate postoperative radiograph showed anatomical reduction of both fractures as well as satisfactory positioning of the hook plate

Among the 1542 clavicle injuries assessed, 15 patients (10 men, 5 women) with bilateral clavicle fractures were treated in our institute (overall incidence of 1.0%). One male patient was excluded because he declined surgical treatment at the authors’ institute after management of life-threatening injuries, and another male patient was excluded because he was not compliant with postoperative follow-up. Thus, this study ultimately included 13 patients with bilateral clavicle fractures (8 men, 5 women) with a mean age of 38.3 ± 15.3 years (range, 18–68 years) (Table 1). The injury mechanisms were car accidents (*n* = 8), machine injury (*n* = 1), bicycle accident (*n* = 1), crashing (*n* = 2), and motorcycle accident (*n* = 1). Ten patients (77.0%) had associated concomitant injuries, among which multiple rib fractures were the most common (7/13), and two patients underwent surgical management of multiple rib fractures. No patients had fractures associated with vascular or neurological injuries. According to the Allman classification, there were 15 simple middle-third fractures, 8 simple distal-third fractures, 2 simple medial-third fractures, and 1 middle-third fracture combined with ipsilateral acromioclavicular joint dislocation (Patient 13). In detail, six patients had bilateral middle-third fractures, four patients had bilateral distal-third fractures, two patients had a medial clavicle fracture associated with a contralateral middle-third clavicle fracture (Fig. 3), and one patient had a middle-third fracture associated with ipsilateral acromioclavicular joint dislocation and a contralateral middle-third clavicle fracture. Only 1 stable non-obviously displaced middle-shaft fracture was treated conservatively; the remaining 25 fractures were fixed with either a superior reconstructive plate, distal clavicle anatomic plate, or hook plate (Fig. 4). All fractures achieved anatomical or otherwise satisfactory bone reduction. One patient (Patient 13) underwent revision surgery of hook plating for treatment of re-dislocation of the acromioclavicular joint in the authors’ institute after primary endobutton fixation of the acromioclavicular joint at a local hospital. Any concomitant injuries were also treated accordingly.

**Table 1:** Patients data and treatment outcomes

No	Gender	Age(years)	Mechanism	Concomitant injuries	Side	Classification	treatment	Follow up(months)	Constant-Murley	DASH	VSA	Complication
1	Female	32	Car accident	Multiple rib fractures	Left	Middle third	Plate	12	100	0	0	None
					Right	Middle third	Plate	12	100	0	0	None
2	Male	18	Car accident	Right subtrochanteric femoral fracture, right humeral shaft fracture, right olecranon fracture, and pelvic fracture	Left	Medial third	Hook plate	88	100	0	0	None
					Right	Middle third	Plate	88	100	0	0	None
3	Male	30	Car accident	Multiple rib fractures, left scapular fracture	Left	Distal third	Plate	13	100	2.5	0	None
					Right	Distal third	Plate	13	100	0	0	None
4	Male	41	Machine injury	None	Left	Middle third	Plate	94	100	0	0	None
					Right	Middle third	Plate	94	100	0	0	None
5	Male	18	Bicycle accident	None	Left	Middle third	Plate	13	100	0	0	None
					Right	Middle third	Plate	13	100	0	0	None
6	Male	53	Crashing	Multiple rib fractures	Left	Middle third	Plate	14	100	0	0	None
					Right	Medial third	Inverted distal clavicle plate	14	96	1.7	0	None
7	Male	32	Motorcycle accident	Maxillofacial fractures, multiple rib fractures, brain injury, left brachial plexus injury	Left	Middle third	Plate	14	34	78.3	3	None
					Right	Middle third	Plate	14	100	0	0	None
8	Female	55	Car accident	Bilateral humeral shaft fracture, left ulnar olecranon fracture, multiple rib fractures	Left	Distal third	Distal clavicle plate	19	92	7.5	0	None
					Right	Distal third	Distal clavicle plate	19	94	5	0	None
9	Male	50	Car accident	C2 vertebral fracture, multiple rib fractures	Left	Middle third	Plate	18	98	2.5	0	None
					Right	Middle third	Plate	18	96	1.7	0	None
10	Female	68	Car accident	Left tibial plateau fracture, right fifth metatarsal fracture	Left	Distal third	Distal clavicle plate	32	96	5		None
					Right	Distal third	Distal clavicle plate	32	96	3.3		None
11	Female	26	Car accident	None	Left	Middle third	Plate	13	96	0	0	None
					Right	Middle third	Conservative treatment	13	100	0	0	None
12	Female	28	Car accident	Right radial shaft fracture, left ulnar olecranon fracture, lumbar fracture	Left	Distal third	Hook plate	42	96	1.7	0	None
					Right	Distal third	Hook plate	42	98	1.7	0	None
13	Male	47	Crashing	Multiple rib fractures, left scapula coracoid process fracture	Left	Middle third fracture + acromioclavicular joint dislocation	Two times of surgery with plate	17	54	35	2	None
					Right	Middle third	plate	17	98	0	0	None

Each patient was followed up for more than 12 months (mean, 29.9 ± 28.5 months; range, 13–94 months), and each patient achieved bone healing on both sides. At the last follow-up, 11 patients had attained excellent shoulder function on both sides and returned to their pre-injury daily activities, and the remaining 2 patients each had unilateral shoulder dysfunction (brachial plexus injury in Patient 7 and complex injury with revision in Patient 13). No complications occurred. Eight patients underwent implant removal after bone union.

## Discussion

Although clavicle fracture is one of the most common fractures, with an incidence of 2.6% to 4.0% of all fractures, its incidence may reach to 5% to 10% in young adults [1]. However, bilateral traumatic clavicle fractures have been very rarely reported [2, 5, 7]. van den Bout [9] reported that the incidence of bilateral clavicle fractures was 0.43% of all clavicle fractures with an overall incidence of 0.011% to 0.017% based on a review of all English-language literature published from 1887 to 2010. Our study showed that bilateral clavicle fractures accounted for nearly 1% of all clavicle fractures, which is a slightly higher incidence than reported by van den Bout [9]. Rowe [12] reported a 1% incidence among 690 clavicle fractures. In this study, we analyzed 15 patients with bilateral clavicle fractures with a male:female ratio of 3:1, and 13 patients were followed up for more than 12 months. To the best of our knowledge, only one report to date has described more cases than in the present study. In 2007, Throckmorton and Kuhn [13] described 10 patients with bilateral middle-third fractures, 4 patients with bilateral distal-third fractures, and 2 patients with bilateral medial fractures among 614 clavicle fractures in 593 patients; however, the authors did not describe the treatments and outcomes of the 16 patients. Other reports that described at least three cases of bilateral clavicle fractures are those by Polaillon [14], who reported eight cases; Marya et al. [15], who reported five cases; Daab et al. [16] and Malgaigne [17], who respectively reported four cases; and Jubel et al. [18] and Lakhotia et al. [6], who respectively reported three cases.

The mechanism of 94% of unilateral clavicle fractures is reportedly a direct blow to the shoulder, whereas that of only 6% is a fall on the outstretched hand [9, 19]. The mechanism of sustaining bilateral clavicle fractures is different from that of a unilateral clavicle fracture. Based on a review of the English-language literature from 1887 to 2010, van den Bout [9] found that the most common causes of bilateral clavicle fractures were a compressive force across both shoulder girdles, direct trauma to both clavicles, direct trauma on one side and indirect trauma by a subsequent fall on the other side, and two sequential episodes of direct trauma to the shoulder. All patients in the present study were injured by high-energy trauma, including eight car accidents, one machine injury, one bicycle accident, two crashes, and one motorcycle accident. Additionally, 10 patients (77.0%) had associated concomitant injuries, the most common of which was chest injury (7/13). With respect to the fracture classification, six patients had bilateral middle-third fractures, four had bilateral distal-third fractures, two had a unilateral medial fracture associated with a contralateral middle-third fracture, and one had a middle-third fracture associated with ipsilateral acromioclavicular joint dislocation and a contralateral middle-third fracture. Bilateral fractures of the medial third of the clavicle reportedly have a high associated mortality rate [9]. In the study by Throckmorton and Kuhn [13], both patients with bilateral medial clavicle fractures died.

In the past, most bilateral clavicle fractures were managed conservatively [20, 21]. However, conservative management of bilateral clavicle fractures was associated with more pain and a high risk of nonunion and shoulder disfunction, rendering the patient incapacitated. Recent reports have advocated operative measures for bilateral clavicle fractures [2, 6, 22]. Operative management can improve patients’ ventilatory function, especially in patients with associated severe chest injuries, because stabilization with osteosynthesis can improve respiratory function and reduce the duration of functional disability. Surgical therapy for bilateral clavicle fractures varies; treatment measures include external fixation [5], pin fixation [23], intramedullary devices [18], and plate fixation [7]. Kirschner wire fixation is not recommended for clavicle fractures because of its high risk of damage to the subclavian neurovascular structures and lungs if hardware migration occurs. Additionally, wire fixation cannot provide adequate stability. External fixation is preferred in cases involving a poor-quality cutaneous environment. Intramedullary fixation cannot provide rotation and length control. Thus, ORIF with a plate is usually recommended for clavicle fractures because it provides rigid fixation with length and angulation control. Among the 13 patients in this study, 25 sides were treated by ORIF with a plate, and the remaining 1 side was treated conservatively. At follow-up, bone union was seen in each side; 11 patients achieved excellent shoulder function on both sides, and the remaining 2 patients had unilateral shoulder dysfunction likely caused by brachial plexus injury and complex injury with revision surgery, respectively. Additionally, all patients were highly satisfied with the treatment.

## Conclusion

Bilateral clavicle fractures are rare and often associated with polytrauma, and they mostly occur in patients who have sustained high-energy trauma. Surgical treatment with plate fixation can result in an excellent outcome in terms of early rehabilitation and return of function, especially when associated severe chest injuries are present.

## Data Availability

The datasets used and/or analyzed during the current study are available from the corresponding author on reasonable request.

## Abbreviation

ORIF: Open reduction and internal fixation
